# Diagnosis of Mucormycosis Using Frozen Section, Histopathology, Culture, and Reverse Transcriptase Polymerase Chain Reaction (RT-PCR) Techniques: A Comparative Study

**DOI:** 10.7759/cureus.56160

**Published:** 2024-03-14

**Authors:** Kala Gnanasekaran Kiruthiga, Anusha Kulkarni, Aparna Joshi, Avinash Pradhan, Sadanand Naik

**Affiliations:** 1 Department of Pathology, King Edward Memorial Hospital, Pune, IND; 2 Department of Biochemistry, King Edward Memorial Hospital, Pune, IND

**Keywords:** frozen, culture, histopathology, rt-pcr, mucormycosis

## Abstract

Mucormycosis usually occurs in immunocompromised patients or those with uncontrolled diabetes. Along the third wave of SARS-CoV-2, an associated angioinvasive opportunistic infection with Mucor, a life-threatening fungal infection, was rampant and emerging. With an increase in the usage of steroids in the COVID scenario, the rate of mucormycosis did take a rapid and alarming increase in King Edward Memorial Hospital, Pune, India. Any delay in the diagnosis and management of the disease was life-threatening. The most conventional methods to diagnose mucormycosis are microbiological culture and histopathology of the tissue. The microbiological culture method plays an important role in the diagnosis of mucormycosis. However, the technique is labour-intensive, taking seven to eight days. Histopathology leads to false-negative reports if the tissue is not biopsied from representative sites. On the other hand, molecular methods are rapid, reliable, and applicable to different body samples, such as tissue, paraffin-embedded tissue blocks, plasma, and urine. We aimed to use a reverse transcriptase polymerase chain reaction (RT-PCR) method to detect Mucor in plasma samples. Due to a lack of availability of fresh samples, nucleic acid was extracted from the tissue sections of 69 cases diagnosed as Mucor by histopathology. These samples were subjected to RT-PCR using the MucorGenius kit (Pathonostics, Maastricht, Netherlands). A total of 57 tissue samples were sent for culture, and 49% of our cases were positive by culture and equally by RT-PCR. There was 80% sensitivity and 76% specificity between culture and PCR techniques. However, the use of blood/plasma for RT-PCR for early diagnosis of mucormycosis will be the method of choice.

## Introduction

Mucormycosis is a life-threatening opportunistic fungal infection with high morbidity caused by filamentous molds belonging to the zygomycetes family. Mucormycosis is a dreadful condition since the fungus is capable of angioinvasion, neural invasion, and bony invasion and can cause direct tissue necrosis and progresses rapidly. Mucormycosis commonly affects immunocompromised patients. During the third wave of SARS-CoV-2-induced COVID-19 pandemic, the world observed an alarming rise in fungal co-infections, especially during the B.1.617.2 (Delta) variant. The prevalence of mucormycosis varied from 0.005 to 1.7 per million population all over the world, while it was 80 times higher in India [1]. Clinically, rhinocerebral mucormycosis had a wide range of presentations, such as nasal block, crusting, facial pain, edema, proptosis, ophthalmoplegia, headache, and fever [2-6]. Untreated rhinocerebral mucormycosis with orbital involvement leading to loss of vision can extend into the cavernous sinus and further cause cerebral invasion causing catastrophic events. This necessitates an early and accurate diagnosis of mucormycosis, which will facilitate early surgical debridement and antifungal medications.

The current diagnosis of mucormycosis is based on histopathological and microbiological studies. In a case report by Chang et al., the diagnosis of mucormycosis was made following the detection of Mucor spp. by histopathological examination with characteristic hyphae of zygomycetes, which were broad, ribbonlike, and predominantly aseptate with wide-angle branching [7]. Special histochemical stains such as periodic acid schiff or Gomori's silver methanamine are used to highlight the fungal hyphae. Mucormycosis is also associated with granulomatous inflammation, and most often has angioinvasion. However, histopathological studies are time-consuming, and many patients progress to losing their vision rapidly. In view of an alarming increase in mucormycosis cases and receiving increased tissue samples from the Department of ENT, we initiated the frozen section procedure in the diagnosis of mucormycosis, especially in highly suspicious cases. This initiative could save the time taken for histopathological processing and allow the surgeon for extended surgery in frozen positive Mucor patients in a single setting. As a second part of the study, we aimed to perform reverse transcriptase polymerase chain reaction (RT-PCR) on blood/ plasma samples or fresh nasal swabs for the diagnosis of Mucor [8-10]. However, when the study was initiated, the number of new cases of mucormycosis was decreasing, and we could not get fresh blood/plasma/nasal swab samples. Hence, the frozen section procedure was done prospectively at the time of diagnosis; however, RT-PCR was performed retrospectively on the tissue blocks of previously diagnosed and confirmed cases of Mucor.

The aims and objectives of the current paper are to compare the efficacy of frozen section and histopathology and to compare the sensitivity and specificity of RT-PCR in the diagnosis of mucormycosis. RT-PCR results are also compared with the culture results wherever available.

## Materials and methods

This is an observational, partly prospective, and partly retrospective study undertaken at the Department of Pathology and Biochemistry, King Edward Memorial (KEM) Hospital, Pune, India. The institutional ethics committee approval (King Edward Memorial Hospital Research Center, approval no. KEMHRC/RVM/EC/2281) was obtained before the start of this research. 1) The prospective part of the study included all biopsy samples of clinically suspected cases of mucormycosis. This amounted to 69 samples. The tissue was subjected to the frozen section procedure, and rapid H&E staining was done. A report of the presence or absence of mucormycosis was informed to the clinicians. Subsequently, more tissue was sent for histopathological examination after complete debridement surgery. Careful examination of the tissue for the presence of hyphae was made in the specimen. Along with it, the tissue was also scrutinized for the presence of other fungal elements, the presence and degree of acute inflammation, necrosis, granulomatous inflammation, angioinvasion, perineural invasion, and bony invasion with osteomyelitis. Inflammation was defined as acute when there were numerous neutrophils. The grading of severity of acute inflammation as mild, moderate, and severe was subjective, and a cut-off of inflammation involving 1/3rd, 2/3rd, and >2/3rd, respectively, of the tissue submitted was used for grading. Granulomatous inflammation was defined as aggregates of epithelioid histiocytes, with or without multinucleate giant cells. Angioinvasion was defined as the presence of fungal hyphae in the wall/lumen of blood vessels. Perineural invasion was defined as the presence of fungal hyphae around neural structures. Osteomyelitis was defined as the presence of necrotic bone trabeculae, associated with neutrophils or lymphocytes or giant cells and fungal hyphae. A short history of the clinical condition of the patient, the presence of comorbidities, and a history of diabetes mellitus/hypertension were noted for the cases available. All the available data were analysed, and results were tabulated. 2) The second part of the study was done prospectively. RT-PCR using the MucorGenius kit (Pathonostics, Maastricht, Netherlands) was carried out on tissue blocks of all patients who were positive for mucormycosis histologically. Of the 69 cases, two cases did not satisfy the quality check of the extracted DNA and were excluded in the second part of the study.

## Results

We have diagnosed a total of 69 cases of mucormycosis in three years (January 2020 to December 2022). The majority of the patients were males (49/69), and 20/ 69 were females. All patients received steroids during SARS-CoV-2 treatment. Fifty-four of them were hospitalized and received oxygen therapy. Twenty-two patients were known diabetics. The patients' age ranged from 26 years to 80 years.

**Figure 1:**
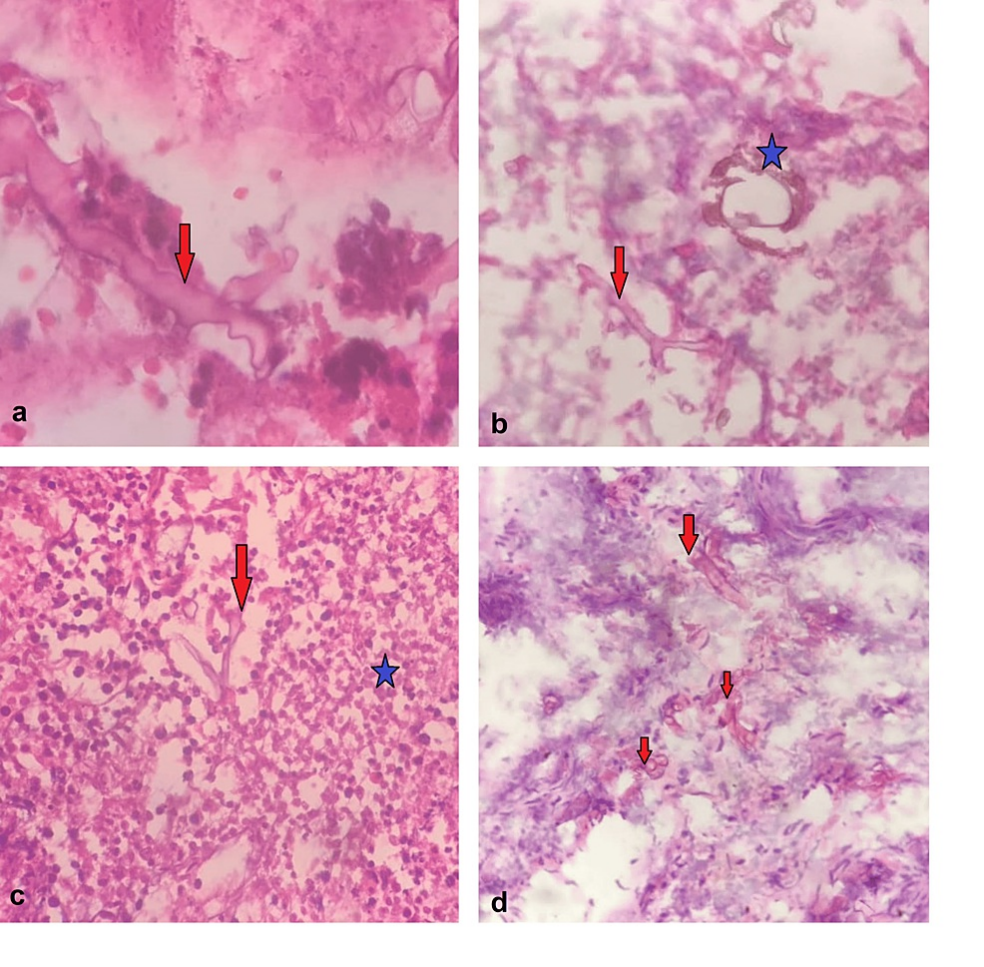
Diffferent appearances of Mucor on frozen sections. a) Hyphae identified by the glassy eosinophilic to basophilic walls (red arrow), H7E stain, 400X. b) Hyphae with right-angled branching (red arrow) and fruiting body (blue star), H&E stain, 200X. c) Hyphae (red arrow) obscured by the numerous neutrophils (blue star), H&E stain, 100X. d) Cross and longitudinal sections of hyphae (red arrow), H&E stain, 200X.

The frozen section procedure was done in 25 cases, of which 12 cases were positive for mucor on frozen section examination, which was further confirmed by a paraffin section study (Figures 1a-1d).

In three cases in which the frozen section was negative, extensive sampling was done in view of high clinical suspicion, and samples from other sites, especially the bones, were sent for the paraffin section, which revealed Mucor. In the remaining 10 cases, both the frozen section and paraffin sections were negative. Thus, the sensitivity and specificity of the frozen section were 100% and 77%, respectively (Table 1).

**Table 1:** Sensitivity and specificity of frozen section for the diagnosis of mucormycosis.

	Histopathology Positive for Mucor	Histopathology Negative for Mucor
Frozen section - Positive for Mucor	12	0
Frozen section - Negative for Mucor	03	10

**Figure 2:**
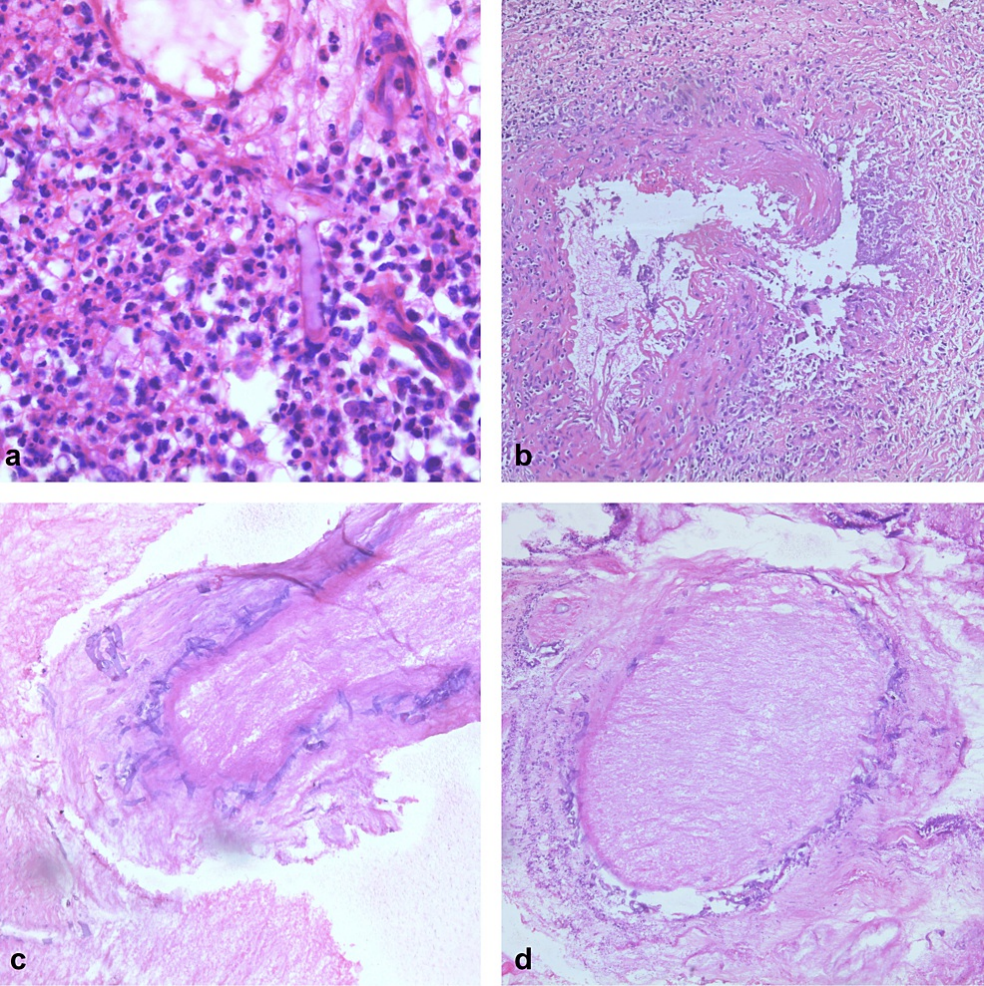
Histopathologic features of mucormycosis. a) Invasion of the wall of the blood vessel by fungal hyphae (red arrow), H&E stain, 200X. b) Circumferential invasion around the nerve by fungal hyphae (red arrow), H&E stain, 200X. c) Invasion of the bone (blue star) by fungal hyphae (red arrow), H&E stain, 200X. d) Granulomatous inflammation with giant cells (red arrow), H&E stain, 200X.

On histopathological examination, most of the cases had severe acute inflammation in 46/69 (67%), moderate inflammation in 5/69 (7%), mild inflammation in 10/69 (14.5%), and no inflammation in 7/60 (10.5%) cases. Granulomatous inflammation was seen in 23/69 (33%) cases (Figures 2a-2b). The histopathological findings are summarized in Table 2.

**Table 2:** Histopathological features in mucormycosis (n=69).

S.No	Histopathological Feature	Number of patients (n=69)	Percentage
1.	Acute inflammation		
	Severe inflammation	46	67%
	Moderate inflammation	5	7%
	Mild inflammation	10	14.5%
	No inflammation	7	10.5%
2.	Granulomatous inflammation	23	33%
3.	Angioinvasion	52	75%
4.	Vascular thrombus	9	8%
5.	Perineural invasion	13	18%
6.	Bone invasion with osteomyelitis	24	34%
7.	Orbital exenteration	7	10%
8.	Mixed infection – Mucor and Aspergillus	9	13%

The paraffin study of Mucor cases revealed angioinvasion in 52 cases (Figure 2a), perineural invasion in 13 cases (Figure 2b), osteomyelitis in 24 cases (Figure 2c), and granulomatous inflammation in 23 cases (Figure 2d). Mixed mucormycosis and aspergillosis were seen in nine cases. Two cases with high suspicion of Mucor did not show hyphae in soft tissue sections; however, on extensive bony decalcification, they were found, confirming a diagnosis of osteomyelitis secondary to mucormycosis.

RT-PCR for mucormycosis was performed on the tissue blocks of 67 out of 69 patients, as two of them did not satisfy the quality check of the extracted DNA. RT-PCR was positive in 33/67 patients (49%); culture was sent only in 57 patients and was positive in 28 (49%). The correlation between RT-PCR and culture was tabulated, and there was statistical significance (p<0.001) (Table 3).

**Table 3:** Correlation between RT-PCR and culture studies.

	RT-PCR Positive (n=30)	RT-PCR Negative (n=27)	P value
Culture positive (n=28)	23	5	<0.001*
Culture negative (n=29)	7	22	

## Discussion

The SARS-CoV-2 infection during the second and third waves of the pandemic created havoc in the world. There was a steep rise in opportunistic fungal infections, including Mucor, Aspergillus, and Candida. Some of the speculations for such a high number of Mucor cases were inherent neutropenia and lymphopenia, uncontrolled diabetes mellitus, use of high-dose corticosteroids, and oxygen therapy, which were used rampantly to treat COVID-19. Mucosal erosions secondary to the aggressive use of steam inhalation or the use of high-flow oxygen was an important postulation [2,5]. Since mucormycosis is a nosocomial fungus and is normally seen in the nasal cavity, immunosuppression and mucosal breach are critical factors that lead to the development of diseases. Contamination from the use of industrial oxygen, low-quality oxygen cylinders, low-quality oxygen piping systems, and ordinary tap water in ventilators are some of the speculated sources from which infection is thought to be acquired [11]. Early accurate diagnosis and prompt treatment significantly reduce mortality and morbidity since the fungus is known for its extensive and rapid angioinvasion and bony invasion [12].

The diagnosis of mucor is mainly by demonstration of the fungus by KOH mount/histopathology/frozen section/culture studies. Microscopically, Mucor is identified as broad, aseptate hyphae with right-angled branching; the cross-sections are large and thick-walled. These structures are usually concealed within the inflammatory infiltrate; however, the refractile nature of these elements is a useful diagnostic clue. Aspergillus hyphae are slender and septate with acute angled branching.

In our study, we have included a total of 69 clinically suspected cases of mucormycosis in three years, between January 2020 and December 2022. Most of them presented with a history of facial pain, dental pain, loss of sensation over the cheek, blurring of vision, diplopia, diminished vision, and headache. A thorough clinical examination was made, and patients were subjected to imaging studies. Later, highly suspicious patients were subjected to surgical debridement involving techniques such as endoscopic nasal and sinus debridement, extended endoscopic nasal and sinus surgery, sublabial/total maxillectomy, endoscopic orbital clearance, and orbital exenteration. The tissue obtained from this procedure was sent for histological and microbiological examination.

On histology, the presence of dense acute inflammation with or without multinucleate giant cells and the presence of tissue necrosis needed a thorough search for fungal elements. Mucor is often camouflaged by the extensive necrotic debris, haemorrhage, and sheets of acute inflammatory cells [11-13]. Freeze artefact, fragmented thick fibres of collagen, refractile crystalline material probably from nasal sprays, cauterized collagen, and mucous strands are some of the mimics of fungal hyphae commonly encountered in frozen sections. These posed significant diagnostic challenges. Sampling the right tissue fragment is of utmost importance because submitting the entire tissue for the frozen section is time-consuming and may be a futile exercise. Grossly, tissue with a greenish/blackish, soft friable appearance as compared to the glistening, whitish to tan soft tissue fragments revealed Mucor in most of the cases. Special stains such as periodic acid schiff (PAS) and Gomori’s methanamine silver (GMS) were not used routinely, since the fungal elements were readily visible on the hematoxylin and eosin stains in 97% of our cases. Severe inflammation and tissue necrosis are clues to the diagnosis of mucormycosis. The frozen section is highly sensitive (100%) and specific (77%) for the diagnosis of mucormycosis, comparable to other studies [14,15]. When the clinical suspicion of Mucor is high and if hyphae are not found in soft tissue sections, in spite of tissue necrosis and inflammation, we recommend sampling the bony tissue for the presence of osteomyelitis associated with mucormycosis. This was seen in two patients in our study.

RT-PCR for mucor was carried out using the MucorGenius kit, and it was positive only in 49% of the histologically confirmed cases. In an ideal situation, RT-PCR should be done on fresh tissue. However, in our study, we performed RT-PCR on the DNA extracted from the tissue blocks. This may have led to inferior quality of nucleic acid material that was amplified in the PCR and hence the results. Culture results were available for 57 cases in the study and were positive only in 49% of them. This may again be due to sampling error, where tissue from other sites (for example a different nasal sinus) was sent for culture. Culture could not be sent to all patients in our study since cost was a limiting factor. The results of RT-PCR when correlated with culture were statistically significant.

Histopathology with or without frozen sections is more sensitive than microbiological studies for the diagnosis of mucormycosis. The frozen section in our setting has helped provide prompt medical treatment to avoid the progression of symptoms, decrease morbidity by extended debridement/maxillectomy at the time of presentation, reduce anaesthesia-associated complications, and significantly reduce the cost of surgery. The correlation between RT-PCR and culture was statistically significant; however, PCR was done on tissue blocks of cases that were positive for Mucor by histopathology. Thus, the correlation between histopathology and RT-PCR could not be carried out. Since RT-PCR was done retrospectively, fresh tissue was not available, and the effect of formalin on the quality of extracted DNA might have been a derogatory factor affecting the results of PCR.

## Conclusions

The frozen section procedure for mucormycosis is very challenging, especially when we encounter artefacts such as crystalline material, ropy collagen, thick mucous strands, and cautery. The different morphological appearances of the fungus on the frozen section need to be mastered by every pathologist. Extensive tissue sampling, sometimes including the bone, is needed to arrive at a correct diagnosis. Tissue necrosis and dense infiltrates of neutrophils are clues to search for fungal hyphae in any patient clinically presenting with nasal block, periorbital pain, and headache. An accurate diagnosis of mucormycosis with diligent, meticulous examination with endurance is rewarding not only to the patient but also to the surgeon and the reporting surgical pathologist. Studies incorporating the examination of Mucor on fresh blood/plasma samples are needed to validate the results, which can be used for a rapid diagnosis of mucormycosis in the future.
